# Whole Exome Sequencing Uncovers Genetic Syndromes Associated with Orofacial Clefts presenting with Limb abnormalities in a Sub-Saharan African cohort

**DOI:** 10.21203/rs.3.rs-8340088/v1

**Published:** 2025-12-15

**Authors:** Edna Tackie, Solomon Obiri-Yeboah, Gideon Okyere Mensah, Tamara D. Busch, Bruce Tsri, Daniel Kwesi Sabbah, Christian Opoku Asamoah, Alexander Acheampong Oti, Gyikua Plange-Rhule, Adebowale A. Adeyemo, Peter Donkor, Azeez Butali, Lord Jephthah Joojo Gowans

**Affiliations:** Kwame Nkrumah University of Science and Technology (KNUST); Kwame Nkrumah University of Science and Technology (KNUST); Kwame Nkrumah University of Science and Technology (KNUST); University of Iowa; Kwame Nkrumah University of Science and Technology (KNUST); Kwame Nkrumah University of Science and Technology (KNUST); Kwame Nkrumah University of Science and Technology (KNUST); Kwame Nkrumah University of Science and Technology (KNUST); Kwame Nkrumah University of Science and Technology (KNUST); National Human Genomic Research Institute; Kwame Nkrumah University of Science and Technology (KNUST); University of Iowa; Kwame Nkrumah University of Science and Technology (KNUST)

**Keywords:** Orofacial clefts, limb abnormalities, genetic syndromes, whole exome sequencing, pleiotropy, polygenic, Sub-Saharan Africa

## Abstract

**Background:**

Orofacial clefts (OFCs) are the most frequent congenital craniofacial anomalies that occur during embryonic development. The incidence is ~1 in 700 live births; it may occur in isolation or with other abnormalities, such as limb deformities. Congenital limb malformations are the second most prevalent birth defect, affecting 1 per 500 to 1000 live births. It can also occur in isolation or as part of a syndrome. This study investigated the genetic aetiology of OFCs co-occurring with limb abnormalities in a Sub-Saharan African cohort.

**Methods:**

Nine unrelated probands with concurrent OFC and limb anomalies were recruited, including one multiplex family involving an affected mother and proband. Whole exome sequencing (WES) was performed at 100X on the DNA samples obtained from affected families, utilising paired-end configuration on the Illumina HiSeq platform. Variant calling utilized the Sentieon workflow. Rare, deleterious variants were identified in accordance with the American College of Medical Genetics and Genomics (ACMG) guidelines on variant classification. *De novo* and other variants predicted as pathogenic were prioritized based on all possible Mendelian inheritance patterns, including variable penetrance and expressivity. Pathway enrichment analysis, protein-protein interactions, and gene expression analysis were undertaken to decipher the biological functions of implicated genes.

**Results:**

All cases were syndromic, presenting with preaxial and postaxial limb anomalies along with other craniofacial features. WES revealed plausible pathogenic variants in pleiotropic genes (*TP63*, *NIPBL*, *MYH3*, *FGFR2*) in four simplex cases. In four other simplex probands, multiple rare variants were identified in developmentally relevant genes (e.g., *RGPD5, FAM90A26, FOXD4L1, FAM170A, DLG1, ANKRD1, TRIM74, TRIM73, PRDM9*) necessary for normal craniofacial and limb development. The multiplex family had two affected individuals (the mother and the proband), both carrying a *TP63* variant, consistent with autosomal dominant inheritance with variable expressivity. Most of the observed variants were *de novo*, with some being novel.

**Conclusion:**

While some cases can be attributed to single-gene syndromes (e.g., NIPBL-associated Cornelia de Lange Syndrome), others may result from multiple co-occurring syndromes. These findings will inform recurrence risk estimates, genetic counselling, and clinical management.

## Background

Orofacial clefts (OFCs) are the most frequent congenital craniofacial anomalies that occur during embryonic development and are mainly characterized by incomplete fusion of the palate, lip, or both. OFCs may manifest in various forms, including bilateral or unilateral, cleft palate (CP) or cleft lip with or without cleft palate, CL/P^1^. It has a global incidence of approximately 1 in 700 live births^2^.

However, the incidence varies significantly across populations, with African populations reporting the lowest incidence (~ 1 in 2000 live births), Europeans reporting an intermediate incidence (~ 1 in 1,000 live births), and Asian populations showing the highest rates, ~ 1 in 500 live births^2,3^. In Ghana, a prevalence of 6.3 per 1000 individuals and a live birth incidence of 1.31 per 1000 have been reported in different communities^4,5^.

A child with CL/P may encounter feeding difficulties, in addition to conductive hearing loss, speech impairments, malocclusion, and aesthetic problems. These physical anomalies can have a significant impact on the psychological and social well-being of the affected individual. Furthermore, the families of these individuals often struggle with societal stigmatization and substantial financial burdens, as the estimated mean lifetime treatment cost is approximately $92,000^6–8^.

OFCs encompass a spectrum, ranging from nonsyndromic, constituting 70% of CL/P and 30% of CP cases, to syndromic clefts, which are associated with other structural anomalies. CP co-occurs in approximately 70% of infants diagnosed with unilateral CL and 85% of those diagnosed with bilateral CL^1,9^.

Syndromic clefts are categorized according to their aetiology into monogenic syndromes, chromosomal syndromes, known teratogen exposures, and uncategorized syndromes that might not fit clearly into any well-defined category due to complex genetic interactions or unidentified aetiological factors^1,10^. The genetic analysis of OFCs poses a significant challenge, as most cases do not strictly conform to the Mendelian inheritance pattern and often occur sporadically as non-familial cases^9^. While the effect size and functionality of pathogenic variants identified through WES are larger compared to loci discovered via GWAS studies, the sporadic nature of most OFC cases continues to complicate genetic variant detection and characterization^11^.

Limb abnormalities encompass a broad range of congenital and postnatal acquired conditions that affect the structure, functionality, or development of the lower and upper extremities. They can be broadly classified into failure of formation of anatomical parts (e.g., amelia, phocomelia, intercalary defects, etc.), failure of differentiation of anatomical parts (e.g., syndactyly, clinodactyly), duplications (e.g., polydactyly), overgrowth and undergrowth (e.g., macrodactyly and brachydactyly), congenital constriction band syndrome, and generalized or systemic skeletal defects^12^. These anomalies may be sporadic and isolated, exhibiting a particular genetic inheritance pattern, or may be associated with specific syndromes. Disruptions during embryonic development, genetic mutations, and environmental factors (e.g., infection and dietary habits) can lead to the development of these anomalies and may impact one or multiple limbs^12^.

Limb defects are the second most common congenital abnormalities in neonates, following congenital heart disease, affecting roughly 4.48 per 10,000 live births globally^13,14^. Syndactyly occurs in approximately 1 in 2,000 to 3,000 live births, whereas polydactyly has a higher incidence, ranging from 1 in 500 to 1,000 live births^15^. The pooled prevalence of congenital anomalies, including limb defects, is approximately 23.5 per 1000 live births in Africa^16^. Clubfoot (talipes equinovarus) is relatively common, with an estimated incidence of 1 per 1000 live births globally and a prevalence of ~ 1.31 per 1,000 live births in the African population^17–19^. Within Ghana, the incidence of clubfoot has been documented at 25.4 per 100,000 live births in the northern population^20^. Syndromic limb malformations carry a substantial mortality risk, with reported survival rates as low as 4% when associated with other organ abnormalities^21^. In Northern Ghana, congenital malformations contribute to an overall neonatal mortality rate of 13.5%, with musculoskeletal system defects ranking as the third most prevalent^22^.

Approximately 10 to 30% of congenital limb defects are syndromic, and ~ 13% are associated with multiple congenital anomalies (MCA), depending on the population^23^. Various exome sequencing and targeted gene studies have implicated several genes in congenital limb defects, including polydactyly (e.g., *GLI3*, *TWIST1, HOXD13*, etc.), syndactyly (*FGFR2*, *GLI3*, *HOXD13, GJA1*, etc.), and ectrodactyly, e.g., *TP63*^15,24^. Several syndromes exemplify the dual presentation of OFCs and limb defects with or without other anomalies. These include the *TP63*-spectrum of disorders, Apert syndrome, Cornelia de Lange Syndrome (CdLS), and VACTERL/ VATER Association^24–27^.

To investigate the genetic architecture of the co-occurrence of OFCs and limb defects, we conducted whole exome sequencing (WES) on nine Ghanaian families, comprising twenty-five individuals in total. This included ten affected individuals, comprising nine probands and one affected mother. Sanger sequencing was subsequently performed to validate the implicated variants. We examined whether the observed co-occurrence arises from monogenic pleiotropy, polygenic burden, or a combination thereof. Our goal was to enhance current understanding of the molecular architecture of syndromic OFCs presenting with limb defects and to elucidate the shared developmental pathways that link the face and limbs.

## Methods

### Study population, participant recruitment, and ethical considerations

The study population consisted of 7 case-parent trios and 2 case-mother dyads, all recruited from the National Cleft Care Centre (NCCC) at Komfo Anokye Teaching Hospital (KATH), Kumasi, Ghana. Each family included a proband diagnosed with OFCs and associated limb malformations, along with one or both of their biological parents.

#### Ethical approval

was obtained from the Institutional Review Board (IRB) at KATH (KATH-IRB/AP/032/20), Kumasi, Ghana. Written informed consent was obtained from participating families prior to the collection of data and samples. All participating families were of Ghanaian descent. Recruited probands presented with CL, CP, or CLP, along with digital anomalies and sometimes other anomalies.

### Sample collection and DNA extraction

Saliva samples were collected using Oragene•DISCOVER saliva tool kits (https://www.dnagenotek.com). Participants who were old enough to spit provided their saliva samples directly, while for those who were not old enough to spit, cheek swab samples were collected.

Genomic DNA was isolated from the buccal swabs and saliva samples using the Oragene protocol at the Human Genetics and Genome (HuGENE) Laboratory at KNUST^19^. The quantity of DNA obtained for each sample was measured using the Qubit Assay (ThermoFisher Scientific, Hampton, USA). XY genotyping using real-time Polymerase Chain Reaction (PCR) was conducted as a quality control measure to verify the genetic sex of study participants^19^. The detailed protocol for DNA processing has been published by Gowans et al. (2016).

### Whole exome sequencing

Whole exome sequencing (WES) was employed to identify genetic variants associated with syndromic OFCs in the recruited Ghanaian trios and dyads. Genomic DNA samples were shipped to commercial sequencing service providers (Azenta Life Sciences, LLC; South Plainfield, NJ, USA), where exome sequencing was conducted.

The genomic DNA underwent fragmentation through acoustic shearing (Covaris S220 instrument). Exonic sequences, flanking intronic regions and UTRs were captured using the Twist Human Comprehensive Exome library preparation procedure (Twist Biosciences, South San Francisco, CA, USA). The fragmented DNAs were purified, end-repaired, adenylated at the 3’ ends, and ligated to adapters. Limited-cycle PCR was used to amplify the adapter-ligated DNA fragments. Validation of the adapter-ligated DNA fragments was performed using Agilent TapeStation (Agilent Technologies, Palo Alto, CA, USA), and quantification was carried out using Qubit 4 Fluorometer (ThermoFisher Scientific, Waltham, MA, USA). The adapter-ligated DNA fragments were subjected to hybridization with biotinylated baits. Streptavidin-coated binding beads were used to capture the resulting hybrid DNAs, after which they were thoroughly washed. The washed captured DNAs were subsequently amplified and indexed using the Illumina indexing primers. Post-captured DNA libraries underwent validation through Agilent TapeStation (Agilent, Santa Clara, CA, USA) and quantification via Qubit 4 Fluorometer and Real-Time PCR (KAPA Biosystems, Wilmington, MA, USA).

Generated sequencing libraries were combined and clustered into multiple sections of a flow cell. Following this, the flow cell was inserted into the Illumina HiSeq instrument, where the samples underwent sequencing in a 2 × 150 bp paired-end (PE) configuration at 100X read depth according to the manufacturer’s instructions. Image analysis and base calling procedures were carried out using the HiSeq Control Software (HCS). The initial raw sequence data in binary base call (.bcl) format produced by the Illumina HiSeq system were transformed into fastq files and de-multiplexed using the Illumina bcl2fastq software. A single mismatch was permitted for the identification of index sequences.

### Bioinformatics analysis for variant calling

**Figure 1:**
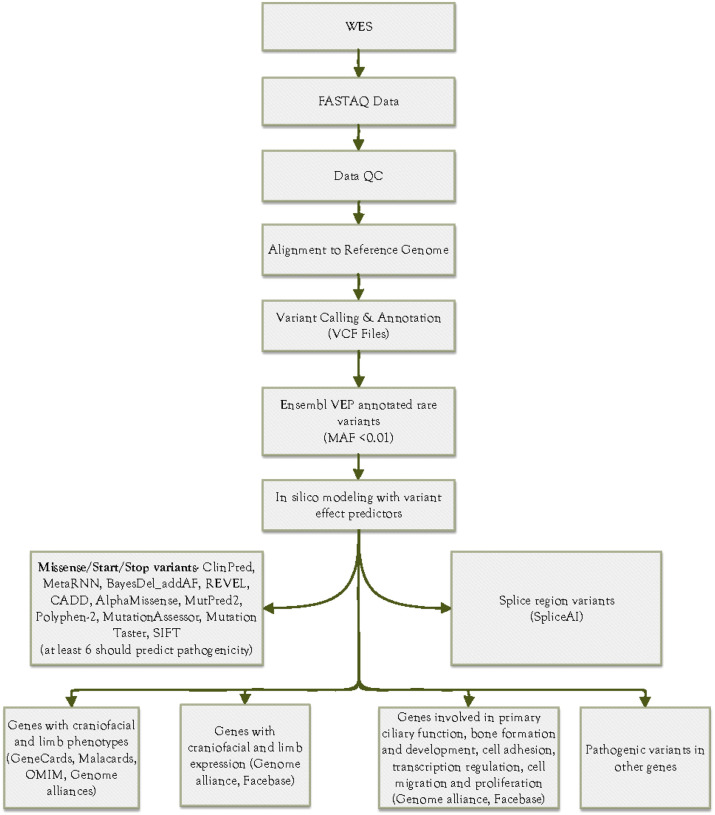
Bioinformatics pipeline for the identification and prioritisation of rare genetic variants potentially associated with craniofacial and limb abnormalities. The variants were prioritized based on pathogenicity predictions, craniofacial and limb phenotypes, gene expression profile, and functional roles in biological processes, based on the ACMG guidelines.

Following WES, raw FASTQ files (Fig. 1) were processed using a standardized bioinformatics pipeline to align reads, call variants, and prioritize candidates. The raw reads generated from WES were quality checked utilising FastQC 0.11.9 and trimmed using Trimmomatic 0.39^29^ to remove sequencing adapters and low-quality bases. The processed reads were aligned to the human reference genome (GRCh38), using the Sentieon 202112.01 workflow^30^. Subsequently, PCR/Optical duplicates were identified and marked, generating BAM files. Single-nucleotide variants (SNVs) and small insertions and deletions (INDELs) were called by employing Sentieon DNAscope algorithm. VCF files generated underwent normalization, including left alignment of INDELs and splitting multiallelic sites into distinct sites, using bcftools v1.13^31^. Subsequently, overlapping transcripts were identified for individual variants, and the potential effects of these variants on the transcripts were annotated utilising the Ensembl Variant Effect Predictor (VEP) v104^32^. For downstream cohort analysis, the most severe impact for each variant was chosen.

### Variant Filtering and Prioritisation Process

Likely pathogenic variants were prioritized (Fig. 1) using the American College of Medical Genetics and Genomics (ACMG) guidelines on variant classification^33^. The variant filtering process was designed to prioritize high-confidence, functionally relevant genetic variants associated with the syndromic OFCs under study. Protein-altering variants, including missense, frameshift insertions/deletions, stop-gained, stop-loss, start-gained, start-loss, and splice region variants with MAF < 0.01 were prioritized. MAF was ascertained using 1000 Genomes Project (https://www.internationalgenome.org/), Exome Sequencing Project (esp.gs.washington.edu/drupal/), and Gnome Aggregation database consortium, gnomAD (https://gnomad.broadinstitute.org/) v4.1. The pathogenicity of all missense variants was determined using both single and meta-prediction tools embedded in dbNSFP v4.9^34^. The tools included ClinPred, MetaRNN, BayesDel_addAF, REVEL, CADD, AlphaMissense, MutPred2, Polyphen-2, MutationAssessor, Mutation Taster, and SIFT (Supplementary Table S1). Missense variants predicted to be pathogenic by at least six out of eleven (i.e., the majority) of the prediction tools were filtered for. The pathogenicity of other variants, such as stop and start gain or loss, was predicted using CADD. Pathogenicity of splice region variants was also determined using SpliceAI embedded in Ensembl VEP. Pathogenic variants in genes associated with craniofacial and limb phenotypes, as well as their associated disorders in humans, were curated using GeneCards (https://www.genecards.org/), Malacards (https://www.malacards.org/), OMIM (https://omim.org/), Genome alliances (https://www.alliancegenome.org/), Mouse Genome Informatics (https://www.informatics.jax.org), and Facebase (https://www.facebase.org/). Newly identified genes involved in biological processes relevant to the phenotypes under study, such as primary ciliary function, bone formation and development, cell adhesion, transcription regulation, cell migration, and proliferation, were curated using Genome Alliance, Mouse Genome Informatics, and Facebase. The co-segregation of candidate variants was assessed to confirm whether identified variants followed Mendelian inheritance patterns (e.g., autosomal dominant or recessive). This was done by verifying the presence of the variant in affected individuals and its absence in unaffected family members, where possible. Given the absence of family history in affected individuals in 8 out of the 9 families, candidate variants were prioritized based on those fitting a model of *de novo* variants or autosomal dominant inheritance with incomplete penetrance. Variants that met the filtering criteria were considered the most likely to cause the observed phenotypes in affected individuals (Fig. 1).

### Sanger sequencing

As a quality control step, two novel candidate variants in *MYH3* (c.2015G > A, p.Arg672His) and *NIPBL* (c.7617_7618del, p.Ser2540ProfsTer21) identified via WES were validated using Sanger sequencing. The exact procedure has been previously published^28^. In summary, primers (Supplementary Table S2) were designed to flank each variant region using Primer3 (https://primer3.ut.ee/). The genomic sequences flanking the variant site were obtained from the UCSC Genome Browser (GRCh38/hg38; https://genome.ucsc.edu/). Each primer pair with closely matched Tm values (within 2°C) was prioritized and validated using BLAT function of UCSC Genome Browser (https://genome.ucsc.edu/cgi-bin/hgBlat) to confirm sequence specificity. I*n-silico* PCR analysis was also performed using the UCSC browser (https://genome.ucsc.edu/cgi-bin/hgPcr) to validate the specificity and production of a single amplicon (300 to 700bp) from the primers.

PCR was used to amplify genomic DNA (4 ng/μL) in 10 μL reaction volumes. Amplification success was verified using 2% agarose gel electrophoresis. Validated amplicons were subjected to sequencing at Functional Biosciences (Madison, WI, USA) using an ABI 3730XL DNA Sequencer. Generated chromatogram data were analysed using PHRED for base calling, PHRAP for assembly, POLYPHRED for variant detection, and CONSED for visualisation, as previously described^19^.

### Structural and evolutionary analysis

For structural analysis of mutant proteins, the potential impact of missense variants on protein structure was evaluated using *in-silico* modelling tools. The protein structures of candidate genes were downloaded from AlphaFold (https://alphafold.ebi.ac.uk/) and visualized using UCSF Chimera v1.14 (https://www.cgl.ucsf.edu/chimera/). The effect of the variant on the protein structures was evaluated using PyMOL v2.3.2 (https://www.pymol.org/).

**Figure 2:**
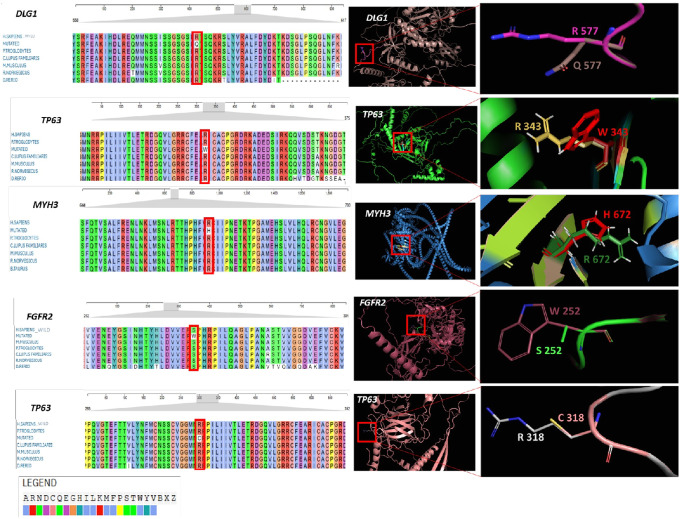
Structural and evolutionary analysis of variants in implicated genes. **(A-E)** Multiple sequence alignment of *DLG1*, *TP63*,*MYH3*, and *FGFR2*across diverse species demonstrated high evolutionary conservation of the wild-type amino acid residue at each position (Highlighted in red boxes). **(F-J)** Structural modelling shows the tertiary protein structure and magnified views of amino acid substitutions, which include replacement of positively charged arginine (pink/yellow/green/white) with polar uncharged glutamine (brown) in *DLG1*, bulky tryptophan (red) in *TP63*, weakly basic histidine (red) in *MYH3*and small polar cysteine (rose) in *TP63*. In *FGFR2*, a small, polar serine (green) is replaced with a bulky aromatic tryptophan (brown).

Evolutionary conservation of identified variants (Table 1) was assessed through multiple sequence alignment of vertebrate orthologs using MAFFT (Multiple Alignment using Fast Fourier Transform) accessed from EMBL-EBI with default parameters^35^. Species included in alignments are indicated in the respective figures (Fig. 2; Supplementary Figure S3). Protein sequences were obtained from the NCBI RefSeq (https://www.ncbi.nlm.nih.gov/refseq/) database and UniProt (https://www.uniprot.org/).

### Pathway enrichment and interaction analysis

Candidate genes obtained after prioritisation were analysed for biological process annotations. The g:Profiler tool (https://biit.cs.ut.ee/gprofiler/gost), a web-based platform, was used to conduct an enrichment analysis of the identified genes against several databases, including Gene Ontology (GO) Biological Process (BP), Molecular Function (MF), and Cellular Component (CC), Reactome, WikiPathways, and Human Phenotype (HP) ontology. g:Profiler performs functional enrichment analysis of gene lists using Fisher’s exact test for over-representation^36^. The analysis was restricted to *Homo sapiens* (human) to ensure species-specific relevance with a significance threshold of p < 0.05. All other parameters were defaulted. The output generated included enriched pathways, their adjusted *P*-value, and the number of overlapping genes from the candidate list. The EnrichmentMap plugin in Cytoscape (v3.10.4)^36^, an open-source platform for visualising complex networks, was employed to cluster and visualize pathway enrichment results as an interactive network. This approach clusters functionally related pathways based on gene set overlap. By doing so, it reduces redundancy, reveals higher-order biological themes, and enables interpretation of enrichment results compared to isolated pathway lists.

### Protein-protein interaction network and hub genes identification

A protein-protein interaction (PPI) network was constructed using the Search Tool for the Retrieval of Interacting Genes (STRING) database v12.0 with an interacting confidence score of > 0.4 (http://string-db.org). The resulting PPI network was exported to Cytoscape for visualisation and further analysis. The CytoHubba plugin, embedded in Cytoscape, was then applied with the maximal clique centrality (MCC) algorithm to identify hub genes^36^, as these highly interconnected nodes are often critical drivers of biological processes.

### Gene expression analysis using mouse models

The gene expression patterns of implicated candidate genes were investigated using the Mouse Genome Informatics (MGI) Gene Expression Database (GXD) v6.24^37^. To refine the search and ensure relevance to the syndromic phenotypes, the curated list of candidate genes was submitted to GXD via the Batch Search interface. A structured filtering approach was implemented to focus on developmentally relevant anatomical regions under the ‘Conceptus’ category. The selected filters include the following terms: 1st branchial arch (mandibular and maxillary components), face mesenchyme, oral region mesenchyme, head surface ectoderm, latero-nasal process, nasal pit, mesenchyme derived from neural crest, and the limb. The ‘heart’ filter was also applied, as two of the probands (Family 2 and Family 4) presented with congenital heart defects. Expression data were retrieved across developmental stages from the earliest post-implantation period through adult stages, with particular emphasis on critical periods of craniofacial (E8.5 to E14.5) and limb (E9.5 to E14.5) morphogenesis. Expression annotations were quantified and visualized via the tissue × gene matrix, with colour intensity corresponding to the number of expression results per anatomical structure. A heatmap was generated using Morpheus^37^, a web-based platform for matrix visualisation and analysis, with qualitative expression levels colour-coded to visualize spatiotemporal gene activity.

## Results

### Clinical presentation of affected individuals

Eight simplex and one multiplex families (Supplementary Figure S1), comprising two dyads and seven trios, with a history of syndromic OFCs presenting with limb abnormalities, were recruited for this study. WES datasets were generated from DNA obtained from case parent trios, except for Families 4 and 6, where the datasets were generated for case mother dyads due to the unavailability of the fathers.

#### Family 1

The proband was a 6-year-old male who presented with a complete cleft palate, ulnar (postaxial) hexadactyly of both hands, mild intellectual disability, speech delay until age 4, mild microcephaly, characteristic facial features (almost V-shaped head with undulating surface), and cephalhematoma (Supplementary Figure S1A).

#### Family 2

The proband was a 5-month-old male who presented with left complete CL, left unilateral talipes equinovarus, microcephaly, a low heart murmur that was suggestive of a heart defect, and large, low-set ears. A follow-up on this family revealed that the child had no neck control at 8 months, suggestive of global developmental delay (Supplementary Figure S1B).

#### Family 3

The proband was a 7-day-old female who presented with bilateral incomplete CLP (right complete CLP plus left complete CP), bifid uvula, ectrodactyly of the right hand (missing/hypoplasia of the third digit with normal development of all other digits), and the fifth digit of the right hand was stiff and could not be bent. The thumb, index, and ring fingers of the right hand were also folded on each other. Clinical evaluation established a diagnosis of ectrodactyly ectodermal dysplasia cleft lip/palate (EEC) based on the presented phenotypes (Supplementary Figure S1C).

#### Family 4

The proband was a 4-month-old female who presented with complete CP, syndactyly of the 2nd and 3rd toes of both feet, severe micrognathia, glossoptosis and breathing difficulty. The proband was re-examined after 8 months, at which point additional phenotypes were observed. She had a short stature, a small and upturned nose, developmental delay, a long philtrum, a thin and downturned upper lip, low-set ears, thick eyebrows, microcephaly, a short fifth finger, arched eyebrows that almost met in the middle, hirsutism, a feeding problem/failure to thrive, long eyelashes, and pulmonary stenosis. The proband was born prematurely during the 8th month of pregnancy with a low birth weight of 1.5 kg. Clinical evaluation established a diagnosis of Cornelia de Lange syndrome based on the presented phenotypes (Supplementary Figure S1D).

#### Family 5

The proband was a 2-weeks-old female who presented with complete CP, bilateral clubfoot, camptodactyly, a characteristic face that causes her to appear to be whistling, microstomia (small puckered mouth), pursed lip, H-shaped scar-like mark extending from the lower lip towards the bottom of the chin, widely-spaced deep eyes, prominent cheeks, struggles to open the mouth, diminished ability to suck, flat philtrum, as well as high-arched and V-shaped palate. Clinical evaluation established a diagnosis of Distal arthrogryposis type 2A (DA2A), also known as Freeman-Sheldon Syndrome (FSS), Supplementary Figure S1E.

#### Family 6

The proband was a female who was 1 month 9 days old at the time of recruitment. She presented with incomplete CP, bulging eyes, and finger and toe syndactyly. These clinical presentations were suggestive of Apert syndrome. The proband was born to consanguineous parents (Supplementary Figure S1F).

#### Family 7

The proband was a 1-week-old male who presented with right complete CLP, unilateral right clubfoot, syndactyly of the 3 middle left toes, left-hand digital hypoplasia with anonychia affecting all fingers except the thumb and right radial clubhand (Supplementary Figure S1G).

#### Family 8

The proband was a 3-day-old male who presented with right complete CLP and hexadactyly, an extra thumb on the right hand (Supplementary Figure S1H).

#### Family 9

The proband was a 2-week-old female. She presented with complete bilateral CLP, ectrodactyly, and ectodermal dysplasia that presented as anhidrosis with impaired thermoregulation and nail dystrophy characterized by thickened nails. These phenotypes were clinically suggestive of EEC syndrome. The proband was born to consanguineous parents. The mother presented with bilateral symbrachydactyly (4th finger), right-hand syndactyly (3–4 fingers) and right foot ectrodactyly (Supplementary Figure S1I).

### Probable pathogenic variants identified by whole exome sequencing

WES analysis of probands with co-occurring OFCs and limb abnormalities unveiled several candidate variants in genes associated with certain developmental pathways and syndromes (Table 1; Supplementary Table S3). These include variants in genes such as *TP63, FGFR2, PRDM9, DLG1, NIPBL* and *MYH3*. Importantly, many of these variants were *de novo*, some of which were novel, suggesting that these variants may be population-specific. As a quality control measure, the variants observed in *NIPBL* and *MYH3* were confirmed with Sanger sequencing (Supplementary Figure S2).

### Structural and evolutionary analysis of implicated variants

The structural and evolutionary analyses of identified missense variants (Table 1) revealed molecular alterations that may underlie the observed phenotypes. The wild-type amino acids are located in highly conserved regions of proteins, with many being perfectly conserved from humans to distant vertebrates such as zebrafish. The structural analyses also revealed a consistent pattern of disruptive alterations across all variants (Fig. 2; Supplementary Figure S3). As a representative example, the R343W substitution in *TP63* occurred in the 8th exon within the DNA-binding domain of the protein, replacing a positively charged arginine with nonpolar tryptophan containing a bulky indole side chain. This substitution eliminates electrostatic interactions between the TP63 protein and the negatively charged DNA phosphate backbone and can destabilize the protein due to unfavourable torsion angles. The *de novo* R672H variant in *MYH3* replaces the positive charge of arginine with a smaller, less charged histidine, potentially weakening ATP binding by reducing electrostatic interactions and decreasing hydrogen bonds critical for the stability of the binding pocket.

### Pathway enrichment analysis, protein-protein interaction network and hub genes identification

**Figure 3:**
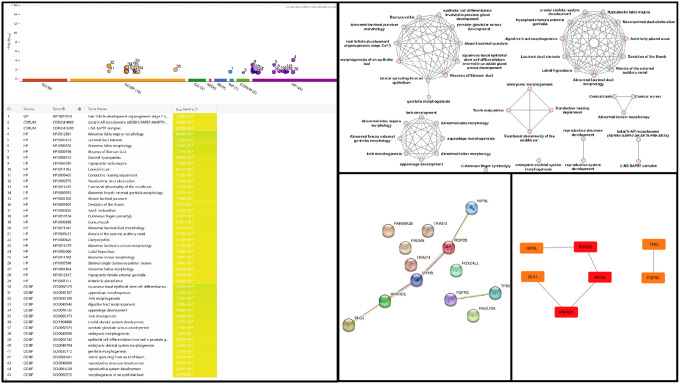
Pathway enrichment and Protein-Protein Interaction Network analysis. (**A**) Significantly enriched biological pathways, processes, and terms obtained from g:Profiler, ranked by −log10 adjusted p-values. (**B**) EnrichmentMap visualisation of the interconnected network of implicated biological pathways, processes and phenotypes. (**C**) PPI network constructed from prioritized genes using the STRING database (interaction score: 0.150). (**D**) Visual representation of hub genes within the PPI network.

Functional enrichment analysis of genes harbouring candidate variants revealed the involvement in several key biological pathways in normal craniofacial and limb development (Fig. 3; Supplementary Figure S4). A highly significant and coherent enrichment related to coordinated developmental processes, specifically those derived from epithelial-mesenchymal interactions, was observed in both Gene Ontology (GO) categories and Human Phenotype Ontology (HP) terms (Fig. 3A and B). A profound statistically significant enrichment for developmental pathways directly related to processes governing limb formation and OFC pathogenesis were observed, including appendage morphogenesis (GO: 0035107, Padj = 1.722×10^− 3^), limb morphogenesis (GO:0035108, Padj = 1.722×10^− 3^), limb development (GO:0060173, Padj = 4.208×10^− 3^), cranial skeletal system development (GO: 1904888, Padj = 1.107×10^− 2^), morphogenesis of an epithelial bud (GO:0060572, Padj = 4.573×10^− 2^) and embryonic morphogenesis (GO: 0048598, Padj = 1.875×10^− 2^). These pathways were complemented by human phenotype ontology (HP) terms, highlighting specific defects such as deviation of the thumb (HP:0009603, Padj = 2.177×10^− 2^) and cutaneous finger syndactyly (HP:0010554, Padj = 2.302×10^− 2^). A significant enrichment was observed for abnormalities in sensory organs that share developmental origins and pathways with the face and limbs, particularly those derived from cranial placodes and neural crest cells^38^. These included lacrimal duct stenosis (HP:0007678, Padj = 1.016×10^− 3^), conductive hearing impairment (HP:0000405, Padj = 1.135×10^− 2^) and nasolacrimal duct obstruction (HP:0000579, Padj = 1.168×10^− 2^). This pattern of phenotypic enrichment corresponds to established clinical features observed in patients who present with both OFCs and limb malformations^39^.

Identification of central regulators (Fig. 3C and D) within the implicated gene set (Table 1) resulted in a PPI network comprising 13 nodes (proteins) and 5 edges (interactions), with an average node degree of 0.769 and a p-value of 0.26 (Supplementary Figure S5). The PPI enrichment p-value indicates that the observed number of connections is not statistically significantly greater than what would be expected by chance for a random set of proteins of the same size and degree distribution from the genome. Given the exploratory nature of our study and to capture potential novel interactions within this specific genetic context, a confidence score threshold of 0.150 was employed. This threshold was selected after the default confidence filter (~ 0.400) yielded only a single interaction, which was deemed insufficient for meaningful network analysis. Despite the lack of statistical enrichment, the analysis identified *RGPD5*, *MYH3*, *ANKRD1*, *DLG1*, *FGFR2*, and *TP63* as the top hub genes (Fig. 3D).

### Spatiotemporal Expression Profiles of Implicated Genes

**Figure 4:**
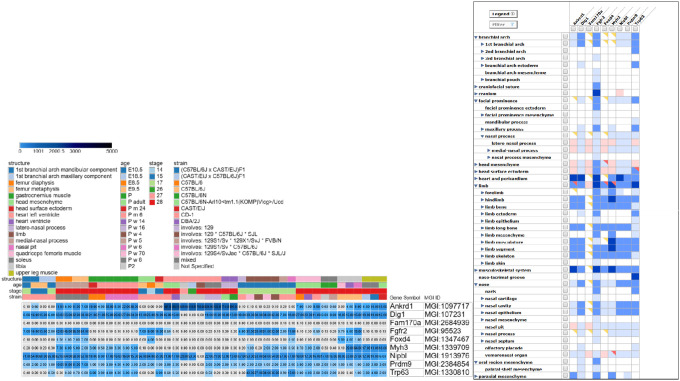
Spatiotemporal gene expression analysis during mouse development. **(A)**Heatmap displaying expression profiles of 9 genes (*Ankrd1*, *Dlg1*, *Fam170a*, *Fgfr2*, *Foxd4*, *Myh3*, *Nipbl*, *Prdm9*, Trp63*) across multiple variables. The top panel shows metadata bars indicating structure types, developmental ages, developmental stages, strain information, and sex designation. Expression levels are gradient colour-coded based on transcripts per million (TPM) from white (0 TPM) through light blue (0.5 TPM) to progressively darker blues, with the darkest blue-black representing values >5000 TPM. Genes are displayed along the y-axis, while embryonic tissues, developmental stages and other variables are shown along the x-axis. Craniofacial tissues (e.g., frontonasal process, palatal shelf mesenchyme, facial prominence) and limb-associated tissues (e.g., limb bud, apical ectodermal ridge, limb mesenchyme, ectoderm, and bone) exhibit overlapping expression profiles across multiple genes. **(B)** Matrix visualisation of gene expression across anatomical structures for nine implicated genes (*Ankrd1*, *Dlg1*, *Fam170a*, *Fgfr2*, *Foxd4*, *Myh3*, *Nipbl*, *Prdm9*, Trp63*). The y-axis lists anatomical structures, including branchial arch components, mesenchyme derivatives, limb structures, and neural crest-derived tissues. The x-axis represents individual genes. Expression data is colour-coded according to the number of expression results annotated: dark blue (present in >50 assays), blue (5–50 assays), and light blue (1–4 assays). Red triangles indicate structures where expression was reported as both present and absent across datasets, yellow triangles represent only absent or ambiguous results in substructures, and empty squares signify the absence of any expression annotations for the respective gene-tissue combination. It should be noted that the visualisation of expression data includes nine of the fourteen genes with variants listed in Table 1, corresponding to the genes for which expression annotations were available in the Mouse Genome Informatics (MGI) database. *TP63 is shown as ***Trp63*** in expression analyses according to mouse ortholog nomenclature

Based on analysis of the Morpheus heat map, nine of the thirteen candidate genes demonstrated distinctive expression patterns associated with specific morphogenetic processes across various mouse strains, developmental stages, and tissue structures (Fig. 4). The other four genes were not represented in the Mouse Genome Informatics (MGI) database utilized for the gene expression analysis. Some genes, including *Dlg1*, *Nipbl*, and *Fgfr2*, exhibited peak expression during early embryonic development, while *Dlg1*, *Nipbl*. *Prmd9* and *Trp63* maintained sustained expression throughout multiple developmental windows (Fig. 4A). Notable *Dlg1*, *Fgfr2*, and *Trp63* expression was observed from Theiler stages (TS) 1 through to 23 in the branchial arches, including ectoderm and mesenchymal components, facial prominence mesenchyme, and palatal shelf, which are regions essential for lip and palate formation. Concurrently, *Dlg1*, *Fgfr2*, *Myh3*, and *Trp63* displayed an overlapping moderate to high expression from TS 15 to 28 in limb mesenchyme, limb ectoderm, and developing skeletal and muscle elements (Fig. 4B).

## Discussion

The study sought to decipher the genetic aetiology of OFCs co-occurring with limb abnormalities, and in some cases, additional phenotypes (Table 1). The co-occurrence of OFCs and limb deformities, though less frequent than isolated OFCs, has significant implications for genetic counselling and clinical management. This co-occurrence is often associated with specific genetic syndromes or different syndromes manifesting concurrently^19^.

WES analysis identified multiple pathogenic variants (Table 1) in affected individuals, some of which have been previously reported and associated with known genetic syndromes. The proband in Family 1 carried two *de novo* variants, one in *RGPD5* and another in *FAM90A26*. *RGPD5* interacts with nuclear proteins, including Ras-related nuclear protein (RAN) and transportin-1^40^. A previous study reported *RGPD5* deletion in an infant with multiple craniofacial and limb malformations, including unilateral complete cleft lip, microcephaly, craniosynostosis, syndactyly, dysmorphic ears, bilateral congenital talipes equinovarus, bilateral radial club hand, and ectrodactyly^41^. The proband in the current study presented with similar phenotypes. *FAM90A26* is expressed in gonad primordial and testicular germline stem cells, with an overexpression of its protein product in the heart (https://www.bgee.org/; https://www.genecards.org/).

Four *de novo* variants were identified in the proband from Family 2 (Table 1), including variants in *FOXD4L1*, *FAM170A*, *DLG1*, and *ANKRD1*. *FOXD4L1* is a forkhead/winged-helix (FOX) transcriptional factor that is crucial for embryogenesis and regulates neural ectoderm by preserving neural precursor cell pluripotency and repressing transcription factors and other genes that drive neural differentiation. These are crucial for FGF and BMP signalling, regulating neural plate patterning and maintaining neural fate^42^. In the expression analysis (Fig. 4), *Foxd4* exhibited an early-onset expression pattern, with craniofacial-specific expression beginning at E7.5 and limited expression in some skeletal elements (femur diaphysis, femur metaphysis, and tibia) between postnatal week 5 to 70. *FAM170A* has been linked with VATER/VACTERL Association^43^, which is defined by the systematic concurrence of vertebral defects (V), anal atresia (A), cardiac malformations (C), tracheoesophageal fistula with or without oesophageal atresia (TE), renal abnormalities (R), and limb anomalies (L)^26^. The proband in the current study presented with CL, talipes equinovarus, global developmental delay, a heart defect, and other abnormalities, which are characteristic of this syndrome. *DLG1*, expressed in both mesenchymal and epithelial cells, plays critical roles in palatal morphogenesis, limb elongation, and spatial patterning. Pathogenic variants in *DLG1* are associated with CLP and disorganized chondrocytes in the sternum, particularly in the proliferative zone^44,45^. Among the implicated genes for this family, *Dlg1* exhibited the second most comprehensive temporal and spatial expression profile, starting at E0.5 and persisting postnatally, with prominent signals in craniofacial tissues (first branchial arch, facial prominences, and palatal shelves) from E8.5 and in limb structures from E10.5. We hypothesize that *DLG1*, a well-established gene associated with clefting and limb defects, contributes to the severe limb and craniofacial phenotypes exhibited by the proband. The *ANKRD1* gene product, cardiac ankyrin repeat protein (CARP), functions as a transcriptional repressor and a sarcomeric component of the titin-binding complex^46^. Pathogenic variants in *ANKRD1* have been implicated in cardiomyopathies and septal defects^46,47^. The proband in the current study also presented with a heart defect characterized by low murmurs, which may result from the pathogenic variant identified in *ANKRD1*. *Ankrd1* is expressed in the heart, starting at E7.5 (TS 11), which coincides with cardiogenic plate formation, as well as craniofacial and limb tissues (Fig. 4). The phenotypic spectrum in the proband suggests a polygenic aetiology, where these four *de novo* variants likely work synergistically to produce the observed craniofacial, limb, and cardiac defects.

Two heterozygous missense variants in *TP63* were identified in individuals from two unrelated families, Family 3 and Family 9, who presented with bilateral cleft lip and palate (CLP), ectrodactyly, and other orofacial and limb malformations. *TP63* functions as a key regulator of ectodermal, limb and craniofacial morphogenesis^48^. Heterozygous variants in *TP63* account for multiple autosomal dominant disorders defined by three key phenotypes: limb defects, ectodermal dysplasia, and facial clefting. At least eight different *TP63*-related syndromes with overlapping phenotypes have been reported^24,48^. *Trp63* exhibits a broad epithelial-enriched expression profile from early embryogenesis to adulthood, with high expression evident in the limb ectoderm and facial prominence ectoderm during their respective windows (E9.5 to E14.5) of outgrowth and fusion (Fig. 4). The first variant (c.1027C > T, p.Arg343Trp) was identified as a *de novo* mutation. A *de novo TP63* variant may represent spontaneous mutagenesis or suggest the presence of undetected germline mosaicism in a phenotypically normal parent^48^. The second variant (c.952C > T, p.Arg318Cys) was inherited from an affected mother. The mother from this consanguineous family (Family 9) presented with bilateral symbrachydactyly, right 3–4 fingers syndactyly, and right foot ectrodactyly, but no orofacial cleft. Both variants observed in *TP63*, occurring within the DNA-binding domain (DBD), are clinically associated with ectrodactyly, ectodermal dysplasia, and cleft lip-palate syndrome 3, EEC3^24^.

A novel heterozygous frameshift variant in *NIPBL* was identified in the proband of Family 4, who was clinically diagnosed with CdLs. Pathogenic variants in *NIPBL* cause CdLS, a complex developmental disorder and the most frequently occurring cohesinopathy. Affected individuals present with impaired growth, abnormal limb development, and developmental impairments^27^. Our spatiotemporal analysis revealed broad early-onset expression of *Nipbl*, with craniofacial and limb expression observed from E8.5 and E10.5, respectively. Cardiac expression began from E9.5, with presence in the primitive heart tube, atria, and ventricles (Fig. 4). This aligns with CdLS clinical phenotypes, where congenital heart defects frequently co-occur with craniofacial and limb malformations^49^. The observed frameshift variant in the current proband may result in a truncated protein, potentially leading to haploinsufficiency of *NIPBL*^49,50^.

A *de novo* heterozygous missense variant in *MYH3* was identified in the proband of Family 5, who presented features descriptive of FSS. Heterozygous variants in *MYH3* cause Sheldon-Hall syndrome (SHS) or FSS, characterized by distinctive facial features, hand and foot contractures, camptodactyly, oropharyngeal defects, and scoliosis^51,52^. The identified variant (c.2015G > A, p.Arg672His) has been previously reported as a recurrent mutational hotspot^52^. Expression analysis revealed early *Myh3* activity localized predominantly to muscle-related structures, including craniofacial muscle compartments and limb muscles. Beyond muscular tissues, *Myh3* was also detected in skeletal elements (Fig. 4).

A heterozygous missense variant in *FGFR2* was identified in the proband from Family 6. The identified variant (c.755C > G, p.Ser252Trp) has been associated with Apert syndrome (AS)^25,53^ and it is detected in ~ 59% of all AS cases, with a strong association with CP^54^. *Fgfr2* was detected in the developing branchial arch mesenchyme and limb bud from their earliest formation stages (E8.5 and E10.5, respectively), with expression persistence throughout major morphogenetic periods and limited expression in the left ventricle of the heart (Fig. 4).

*De novo* heterozygous stop-gained variants in *TRIM73* and *TRIM74* were observed in the proband from Family 7. These genes have been associated with Williams-Beuren syndrome (WBS), which results from abnormal homologous recombination and unequal crossing-over between tandem segments containing *TRIM50*, *TRIM73*, and *TRIM74*^55^. The TRIM proteins function as E3 ubiquitin ligases, conferring substrate specificity to ubiquitin-proteasome complexes and thereby affecting nearly all cellular processes^56^. *TRIM73* and *TRIM74* share a high degree of similarity (~ 99.8%), indicating redundant paralogs with overlapping functions in developmental processes, potentially providing functional compensation through genetic redundancy. WBS is defined by unique dysmorphic craniofacial features, supravalvular aortic stenosis (SVAS), hypertension, depressed nasal bridge, intellectual disability, premature ageing of the skin, broad forehead, infantile hypercalcemia, and tooth defects^57,58^. Interestingly, the phenotypes of this syndrome were palpable in the current proband, who presented with right complete CLP, right clubfoot, left toes 2–4 syndactyly, left-hand digital hypoplasia with anonychia affecting all fingers except the thumb, and right radial clubhand. We hypothesize that the involvement of TRIM proteins in cellular processes, such as apoptosis and cell proliferation^59^, may underlie the observed phenotypes. However, further functional genomics experiments are warranted to confirm these observations.

We also identified a novel *de novo PRDM9* frameshift variant (c.2272_2273insTG, p.Arg758LeufsTer182) in the proband from Family 8. *PRDM9* is specifically expressed in germ cells during meiosis, where its methyltransferase activity increases H3K4me3 at recombination hotspots^60^, thereby acting as a crucial determinant of meiotic recombination in both murine and human models^61^. *PRDM9* has been implicated in Smith-Magenis Syndrome (SMS), an autosomal disorder characterized by OFCs, depressed nasal bridge, hand polydactyly, toe syndactyly, clinodactyly of the 5th finger, micrognathia, etc^43^. The current proband presented with complete CLP and hand hexadactyly, characteristic of SMS. Population-specific recombination patterns associated with *PRDM9* alleles may contribute to disease-causing genomic rearrangements, particularly in individuals of West African ancestry^62^. In our spatiotemporal analysis, *Prdm9* showed expression in craniofacial tissues (branchial arch, facial prominence, head mesenchyme, head surface ectoderm) from E8.5 and limb structures from E10.5 through to adulthood (Fig. 4), suggesting a role for this gene in craniofacial development.

A consistent pattern of disruptive molecular alteration and loss of conserved amino acid residue was observed for all studied variants in the structural and evolutionary analyses (Fig. 2; Supplementary Figure S3). Multiple sequence alignment showed that all the wild-type amino acids subject to mutation were highly conserved across vertebrate species. The identified substitutions consistently resulted in detrimental structural changes, including loss of critical electrostatic interactions, introduction of steric clashes, disruption of hydrogen bonding networks, and alteration of binding pocket geometries.

Pathway enrichment analysis (Fig. 3A) revealed that the implicated genes are significantly involved in appendage morphogenesis, cranial skeletal system development, and embryonic morphogenesis pathways in craniofacial and limb development. Human phenotype ontology (HP) terms also revealed enrichment for phenotypes such as nasolacrimal duct obstruction, cutaneous finger syndactyly, and deviation of the thumb, corresponding to the established clinical features observed in patients who present with both OFCs and limb malformations^39^.

The EnrichmentMap analysis generated a network of 46 nodes and 140 edges, organized into clusters and individual nodes (Fig. 3B). The first cluster centred on epithelial and glandular development, indicating that disrupted epithelial-mesenchymal interactions underlie palatal, limb, and glandular malformations (Doshi and Patil, 2012). The second cluster focused on craniofacial development and urogenital morphogenesis. The third cluster was dominated by appendage and limb morphogenesis, co-enriched with abnormalities of the external genitalia, which indicate shared developmental pathways.

The protein-protein interaction network (Fig. 3C) revealed limited connectivity among the candidate genes, with only 5 interactions identified among the 13 proteins (Supplementary Figure S5). Despite the sparse network structure (PPI enrichment p-value = 0.26), *RGPD5*, *MYH3*, *ANKRD1*, *DLG1*, *NIPBL*, *FGFR2*, and *TP63* emerged as key hub genes (Fig. 3D) with established roles in pathways fundamental to embryogenesis, specifically craniofacial and limb morphogenesis.

The extrapolability of the findings of the current study is limited by its small sample size. Again, our inability to obtain paternal samples in two families hindered our ability to determine whether certain variants are *de novo*. Lastly, the study focused on the analysis of WES datasets to decipher single nucleotide variants (SNVs) and indels in the aetiology of the conditions under study. As a result, potentially pathogenic non-coding and structural variants could have been missed.

## Conclusion

In conclusion, WES analysis of nine Ghanaian families presenting with OFCs and limb abnormalities, and in some instances other abnormalities, identified probable pathogenic genetic variants, including novel and population-specific mutations in various genes, confirming the genetic heterogeneity of OFCs co-occurring with limb malformations. These variants support a model in which both pleiotropic, monogenic, and polygenic mechanisms contribute to phenotypic variations. Future studies incorporating larger, more diverse cohorts and functional validation approaches will help expand variant discovery, clarify biological mechanisms, and improve genetic counselling and clinical management of syndromic OFCs.

## Supplementary Material

Supplementary Files

This is a list of supplementary files associated with this preprint. Click to download.

- Additionalfile1.docx
- AdditionalFile2.docx
- AdditionalFile3.docx
- AdditionalFile4.docx
- AdditionalFile5.docx
- AdditionalFile6.docx
- AdditionalFile7.docx
- AdditionalFile8.docx

## Figures and Tables

**Table 1:** Pathogenic variants identified through whole exome sequencing in nine affected probands.

Family ID (Sex)	Gene (Inheritance)	Genomic Co-ordinates	Genotype (*Zygosity*)	HGVSc	HGVSp	No. of Tools Predicting Pathogenicity
1 M(XY)	*RGPD5 (De Novo)*	chr2:109837829 (rs1553471918)	*Het*	c.4708G > A	p.Gly1570Arg	7
	*FAM90A26 (De Novo)*	chr4:9173181 (rs1450748908)	*Homo*	c.10del	p.Cys4ValfsTer12	N/A
2 M(XY)	*FOXD4L1 (De Novo)*	chr2:113499585 (rs201655302)	*Het*	c.329A > C	p.Tyr110Ser	9
	*FAM170A (De Novo)*	chr5:119634539 (rs754719389)	*Het*	c.791T > C	p.Met264Thr	7
	*DLG1 (De Novo)*	chr3:197090942 (rs769502806)	*Het*	c.1730G > A	p.Arg577Gln	6
	*ANKRD1 (De Novo)*	chr10:90917812 (rs773773073)	*Het*	c.472C > T	p.His158Tyr	11
3 F(XX)	*TP63 (De novo)*	chr3:189868614 (rs886041251)	*Het*	c.1027C > T	p.Arg343Trp	11
4 F(XX)	*NIPBL* (Unknown)*	chr5:37059093 (*Novel*)	*Het*	c.7617_7618del	p.Ser2540ProfsTer21	N/A
5 F(XX)	*MYH3 (De novo)*	chr17:10641317 (rs121913617)	*Het*	c.2015G > A	p.Arg672His	9
6 F(XX)	*FGFR2* (Unknown)*	chr10:121520163 (rs79184941)	*Het*	c.755C > G	p.Ser252Trp	10
7 M(XY)	*TRIM74 (De Novo)*	chr7:72961358 (rs199887265)	*Het*	c.487C > T	p.Arg163Ter	CADD score of 35
	*TRIM73 (De Novo)*	chr7:75403732 (rs199982097)	*Het*	c.487C > T	p.Arg163Ter	CADD score of 35
8 M(XY)	*PRDM9 (De Novo)*	chr5:23527360 (*Novel*)	*Homo*	c.2272_2273insTG	p.Arg758LeufsTer182	N/A
9 F(XX)	*TP63* (*Maternal inheritance*)	chr3:189867902 (rs1205536026)	*Het*	c.952C > T	p.Arg318Cys	11

* Paternal samples of Families 4 and 6 were unavailable; however, the identified variants for these two families were absent in the mothers. *Het*: heterozygous; *Homo*: Homozygous.

## Data Availability

The whole exome sequencing (WES) dataset reported in this article can be accessed through FaceBase Consortium (https://www.facebase.org) under controlled access, with an accession number 94-D420 (Gowans LJJ, 2025). The informed consent obtained from participants only permits sharing the WES dataset under controlled access. The data and materials that support the findings of this study are available from the corresponding author upon reasonable request. The data and materials that support the findings of this study are available from the corresponding author upon reasonable request.

## Abbreviations

*ANKRD*: Ankyrin Repeat Domain
BMP: Bone morphogenic protein
CdLS: Cornelia de Lange syndrome
CL: Cleft lip
CL/P: Cleft lip with or without cleft palate
CLP: Cleft lip and palate
CP: Cleft palate
DA2A: Distal arthrogryposis type 2A
*DLG1*: Discs Large MAGUK Scaffold Protein 1
DNA: Deoxyribonucleic acid
EEC: Ectrodactyly ectodermal dysplasia cleft lip/palate
*FAM170A*: Family with Sequence Similarity 170 Member A
*FAM90A26*: Family with Sequence Similarity 90 Member A26
FGF: Family with Sequence Similarity 90 Member A26
*FOXD4L1*: Forkhead Box D4 Like 1
FSS: Freeman-Sheldon syndrome
*GLI3*: GLI Family Zinc Finger 3
GWAS: Genome-wide association studies
HuGENE: Human Genetics and Genome
KATH: Komfo Anokye Teaching Hospital
KNUST: Kwame Nkrumah University of Science and Technology
MAGUKs: Membrane-associated guanylate kinase homologs
*MYH3*: Myosin Heavy Chain 3
*NIPBL*: Nipped-B-like protein
OFCs: Orofacial clefts
*PRDM9*: PR domain-containing 9
*RGPD5*: RANBP2-Like and GRIP Domain Containing 5
*SHS*: Sheldon-Hall Syndrome
*TP63*: Tumor protein p63
*TRIM*: Tripartite motif
WES: Whole exome sequencing
WGS: Whole genome sequencing
